# Providing Impetus, Tools, and Guidance to Strengthen National Capacity for Antimicrobial Stewardship in Viet Nam

**DOI:** 10.1371/journal.pmed.1001429

**Published:** 2013-05-07

**Authors:** Heiman F. L. Wertheim, Arjun Chandna, Phu Dinh Vu, Ca Van Pham, Phong Dai Thi Nguyen, Yen Minh Lam, Chau Vinh Van Nguyen, Mattias Larsson, Ulf Rydell, Lennart E. Nilsson, Jeremy Farrar, Kinh Van Nguyen, Håkan Hanberger

**Affiliations:** 1 Oxford University Clinical Research Unit, Hanoi, Viet Nam; 2 South East Asia Infectious Disease Clinical Research Network, Ho Chi Minh City, Viet Nam; 3 National Hospital for Tropical Diseases, Hanoi, Viet Nam; 4 Hospital for Tropical Diseases, Ho Chi Minh City, Viet Nam; 5 Linköping University, Linköping, Sweden; 6 Department of Infectious Diseases, Östergöland, County Council of Östergöland, Linköping, Sweden

## Abstract

Heiman Wertheim and colleagues describe the launch and impact of VINARES, an initiative to strengthen antimicrobial stewardship in Vietnam.

**Summary Points**

- Antimicrobial stewardship has been difficult to implement globally, and in emerging economies it is usually absent or inadequate.
- Implementation of antibiotic stewardship programmes need not wait for “perfect local data” or funding. There is often sufficient local expertise to start a programme in the absence of additional resources. No immediate impact on resistance levels should be expected.
- Antibiotic resistance is partly the result of a dysfunctional health system. Long-term commitment is necessary to improve healthcare infrastructure in order to establish a successful antibiotic stewardship programme and reduce resistance rates.
- Healthcare professionals, preferably “respected” figures, should coordinate stewardship programmes. The programmes should be contextualised and compatible with local practices to encourage engagement and compliance of all healthcare workers.

## Antimicrobial Resistance in Emerging Economies: An Urgency to Intervene

Antimicrobial resistance is a major global health threat. In the European Union an estimated 25,000 deaths occur annually secondary to multi-drug-resistant infections [1]. No reliable estimates for developing countries exist, but figures are likely to be higher. Strategies to contain antimicrobial resistance were comprehensively set forth by the World Health Organization (WHO) in 2001 [2]. However, implementation in low- and middle-income countries, where the need for effective antimicrobials is greatest, has thus far proved problematic [3],[4].

In Viet Nam, where resistance rates are among the highest in Asia [5], the challenge is urgent and great [6]. A large population, high infectious disease burden, and unrestricted access to antimicrobials make Viet Nam a hotspot for the emergence of drug resistance [7]. Adequate legislation to tackle antimicrobial resistance in Viet Nam already exists [7]–[9], but a lack of resources prevents effective policy enforcement [7].

Growing global consensus that antimicrobial resistance must be tackled provides opportunity to intervene. Interventions must account for limited resources available in many regions with the highest resistance burdens. In response to this we launched the Viet Nam Resistance (VINARES) project, a capacity-building initiative designed to strengthen antimicrobial stewardship in Viet Nam. The framework of VINARES may be transferable to other settings, and emerging economies in particular.

## Conception of VINARES

In 2010 a situation analysis of antibiotic use and resistance in Viet Nam identified sub-optimal infection control, inadequate laboratory diagnostic capacity, and inappropriate antibiotic therapy as important drivers of bacterial resistance [7]. Parallels exist with the priorities in the WHO antimicrobial resistance policy package issued on World Health Day, 2011 [10]. However, a dysfunctional health system prevents translation of this research into action [4],[7].

**Figure 1:**
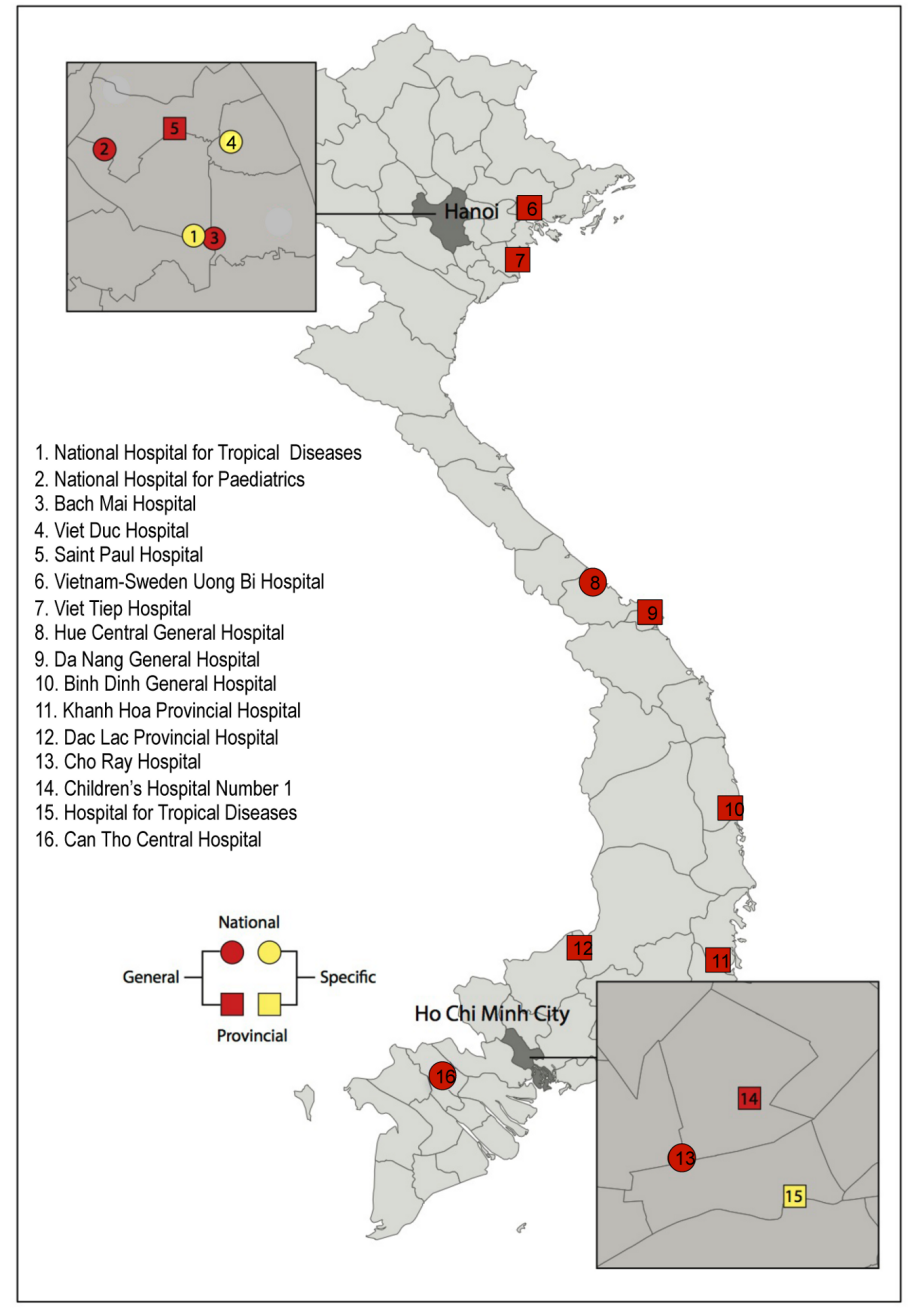
Location, speciality, and type of the 16 participating hospitals in the VINARES project.

VINARES aims to provide impetus and tools to support development of an effective antimicrobial stewardship programme for Viet Nam and thus bridge the “know–do” gap that often plagues implementation of health initiatives in emerging economies [11]. It is a partner-driven collaboration, conceived by Vietnamese healthcare professionals, the Oxford University Clinical Research Unit and Wellcome Trust Major Overseas Programme in Viet Nam, and Linköping University, Sweden. Together we invited 16 hospitals to participate (Figure 1). Practising Vietnamese healthcare professionals coordinate each arm of the project, which address the following areas: (1) infection control and healthcare-associated infections (HAI), (2) antibiotic consumption, and (3) microbiological analysis and reporting capacity.

### Infection Control and Healthcare-Associated Infections

With the new antibiotic development pipeline running dry, infection *prevention* is increasingly important [12]. Infection control is particularly problematic in Vietnamese intensive care units (ICUs) [6]. As measures of HAI control are a good surrogate for overall infection control [13], assessing the HAI burden in ICUs is an effective way to identify areas for improvement.

HAIs are assessed by monthly point prevalence surveys (PPSs) in the ICUs of participating hospitals. The surveillance design is similar to methodologies implemented by the European Centre for Disease Prevention and Control [14], contextualised to the Vietnamese setting. Data are entered into European Centre for Disease Prevention and Control software (HELICSwin), which has been translated into Vietnamese. Traditional HAI risks are recorded, as well as those specific to the Vietnamese healthcare system, for example, the hands-on involvement of family in patient care, the large numbers of staff and students accessing the ICU, and the high prevalence of tetanus patients requiring extended periods of invasive mechanical ventilation. Preliminary data show that 75% of ICU patients have family involved in caretaking. The aim is to emphasise the importance of surveillance, identify common problems in ICUs, and establish priorities accordingly. To date, data on over 1,300 patients in Vietnamese ICUs have been collected.

### Antibiotic Consumption

Antibiotic use—appropriate and inappropriate—drives resistance [15]. We have designed a simple database to help pharmacists calculate monthly antibiotic consumption in defined daily dosages. In addition to reducing the *number* of prescriptions, improving prescribing *rationale* is essential [10]. For each patient receiving antimicrobials at the time of the PPS we collect information on route of administration, dose, and prescription indication. Inappropriate antimicrobial use, particularly for medical and surgical prophylaxis, is common in Viet Nam [16].

### Microbiological Analysis and Reporting Capacity

Appropriate antibiotic use requires a hospital's healthcare professionals to act in unison. It cannot be achieved if physicians prescribe idiosyncratically. Standard treatment guidelines (STGs) are essential to coordinate prescribing, but their formulation requires reliable data on causative organisms and antibiotic susceptibility. To deliver this, enhancing laboratory capacity is crucial [17].

We have provided each laboratory with a computer, surveillance database software (WHONET), current antibiotic susceptibility testing guidelines from the Clinical and Laboratory Standards Institute translated into Vietnamese, and American Type Culture Collection reference strains for internal quality control. Many Vietnamese laboratories perform susceptibility testing to drugs that is not indicated, often giving discrepant, erroneous results and wasting resources [18]. The training and materials provided by VINARES aim to remedy this.

We have applied for all laboratories to participate in an external quality assurance programme (United Kingdom National External Quality Assessment Service). Laboratories can send important resistant strains (vancomycin-resistant *Staphylococcus aureus*, vancomycin-resistant enterococci, and carbapenem-resistant enterobacteria) to a reference laboratory for confirmation testing. If verified, dissemination of important information to the Vietnamese and international scientific community is possible. Furthermore, feedback and training are given if the resistance phenotype is not confirmed. In several laboratories we observed an improbably high rate of vancomycin-resistant *S. aureus*. We found this high rate to be due to erroneous testing methodology and gave recommendations to correct this.

## Implementation of VINARES

In September 2012, 110 delegates from participating hospitals and the Viet Nam Ministry of Health attended inaugural workshops. Delegates included hospital directors, clinicians, infection control doctors, microbiologists, and pharmacists. Day one of the workshop provided a forum for each hospital to present important aspects of infection control, HAIs, antibiotic consumption, and antimicrobial stewardship at their respective hospitals. A key objective of VINARES is to motivate change by sharing information and learning from each other. The workshops were the first stage in this process.

Day two was primarily for training. Participants attended focus groups: use of antibiotic consumption software (pharmacists), resistance surveillance and training in microbiological techniques (microbiologists), and introduction to the PPS and HELICSwin (infection control and ICU doctors). Discussions stimulated modifications to the protocol. For example, a more detailed assessment of family involvement in patient care was included, and discrepancies between European brand names and the Vietnamese formulary were addressed. These modifications highlight the importance of consulting with local stakeholders when implementing an initiative such as VINARES.

**Figure 2:**
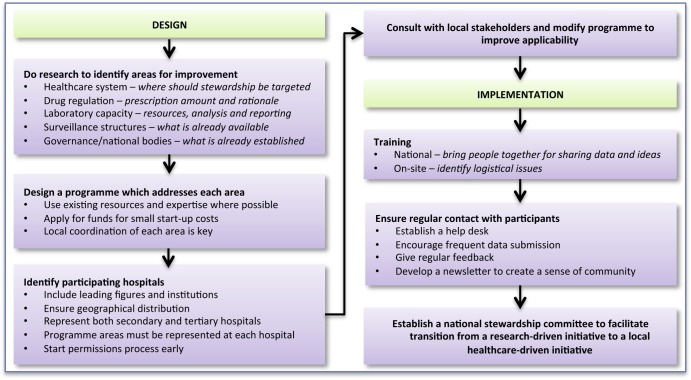
Bridging the “know–do” gap: design and implementation of an antimicrobial stewardship programme in an emerging economy.

After the workshops, implementation teams visited each hospital to deliver two computers containing databases and protocols, assist with the first PPS, and configure a functioning WHONET database for each laboratory. A dedicated help desk was established, which hospitals could contact by E-mail. Figure 2 provides a framework for designing and implementing a stewardship programme in a resource-limited country.

## Challenges along the Way

### Data Quality

Effective stewardship requires reliable data. However, in resource-limited settings poor data are initially acceptable as they identify where to improve. Emphasis is on encouraging stewardship and building capacity.

Modification of the PPS following suggestions from participants and on-site training in surveillance methodologies has reduced ambiguity and encouraged uniform cross-site data collection. Site visits have ensured a minimum operating standard of laboratories. VINARES coordinators perform monthly data quality checks.

### Permissions

This project required permission from the Viet Nam Ministry of Health and approval from the Institutional Review Board of the National Hospital for Tropical Diseases. This process required close monitoring to ensure it progressed. Good relationships and patience were key. Another challenge was acquiring import permits for external quality assurance panels containing live bacteria. The government requested that we reveal the identity of the bacteria; however, the nature of proficiency panels is that their identity should remain unknown. This required further meetings to explain the purpose of external quality assurance.

### Ownership of Data

Ownership of data is contentious, particularly for sensitive issues such as HAIs and infection control. Building trust is essential to achieve sharing of data, upon which interventions can be developed. VINARES aims to encourage sharing of data between institutions, but this is understandably approached with caution. However, most hospitals have been willing to share data.

## Standard Treatment Guidelines

Early in 2013 local stakeholders from participating hospitals met to draft STGs for important ICU infections, including pneumonia (community- and hospital-acquired) and meningitis. Data submitted from VINARES were reviewed and used to begin formulation of the first evidence-based STGs for Viet Nam. Using national surveillance data as a primary source will permit current and contextualised STGs, which we aim to make readily available throughout Viet Nam with the expected launch of the national stewardship website in June 2013.

## After VINARES

**Figure 3:**
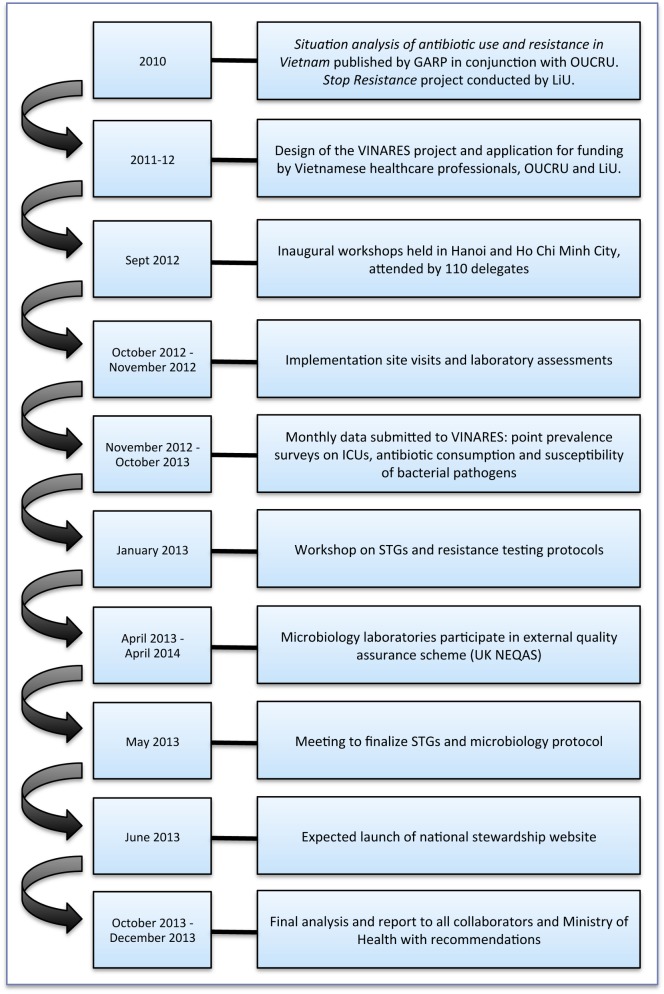
A timeline to illustrate the conception and implementation of the VINARES project. GARP, Global Antibiotic Resistance Partnership; LiU, Linköping University; OUCRU, Oxford University Clinical Research Unit; UK NEQAS, United Kingdom National External Quality Assessment Service.

VINARES is a capacity-building initiative designed to equip hospitals with the tools to perform self-sufficient antimicrobial stewardship for the long term. Hospitals submit monthly data on antibiotic consumption, infection control surveillance, and susceptibility of bacterial pathogens. We provide hospitals with regular reports, comparing their results to the average performance of all hospitals. We hope personalised feedback, including newsletters, will motivate appropriately targeted service change. Figure 3 illustrates a timeline for the VINARES project.

Hospitals will receive a full year of support to establish antimicrobial stewardship programmes. If local leaders and physicians are convinced that patient care has improved, they will be motivated to maintain the surveillance structures established during that year.

VINARES addresses almost all hospital-related priorities in the WHO policy package on antimicrobial resistance (Table 1) [10]. Its purpose is to bridge the gap between policy and action that has hampered antimicrobial stewardship in low- and middle-income countries [11]. Through VINARES, 16 hospitals have committed to improving antimicrobial stewardship in Viet Nam for the next 12 months. In “training the trainers”, we hope that other hospitals, in Viet Nam or abroad, will follow, with support from one of these 16 hospitals.

**Table 1:** Drivers of antibiotic resistance, hospital-related WHO policy package priorities, and how these are met by the VINARES project [10].

Resistance Driver	WHO Priority	VINARES Project
Fragmented, non-comprehensive action	Comprehensive, financed, concerted national action	Doctors, microbiologists, and pharmacists brought together; data from 16 hospitals collated and fed back to hospitals for concerted action
Paucity of data	Strengthen surveillance	Surveillance tools installed, and data submitted to central database each month
Lack of STGs	Promote rational use of medicines	Create a committee to use primary data and existing literature to formulate the first evidence-based STGs for Viet Nam
Weak infection prevention and control	Enhance infection prevention and control	PPSs to identify problematic areas in infection control and HAIs, and establish priorities accordingly
Inadequate laboratory capacity	Strengthen laboratory capacity	Each laboratory provided with computer, translated current susceptibility testing guidelines, external quality assurance, and surveillance database software

## Abbreviations

HAI: healthcare-associated infection
ICU: intensive care unit
PPS: point prevalence survey
STGs: standard treatment guidelines
VINARES: Viet Nam Resistance
WHO: World Health Organization
